# Mobile Health Intervention to Close the Guidelines-To-Practice Gap in Hypertension Treatment: Protocol for the mGlide Randomized Controlled Trial

**DOI:** 10.2196/25424

**Published:** 2021-01-25

**Authors:** Kamakshi Lakshminarayan, Thomas A Murray, Sarah M Westberg, John Connett, Val Overton, John A Nyman, Kathleen A Culhane-Pera, Shannon L Pergament, Paul Drawz, Emily Vollbrecht, Txia Xiong, Susan A Everson-Rose

**Affiliations:** 1 Division of Epidemiology & Community Health School of Public Health University of Minnesota Minneapolis, MN United States; 2 Division of Biostatistics School of Public Health University of Minnesota Minneapolis, MN United States; 3 Department of Pharmaceutical Care & Health Systems College of Pharmacy University of Minnesota Minneapolis, MN United States; 4 Fairview Health Services Minneapolis, MN United States; 5 Division of Health Policy and Management School of Public Health University of Minnesota Minneapolis, MN United States; 6 SoLaHmo Partnership for Health and Wellness Minneapolis, MN United States; 7 Minnesota Community Care Saint Paul, MN United States; 8 Division of Renal Disease and Hypertension Medical School University of Minnesota Minneapolis, MN United States; 9 Department of Medicine and Program in Health Disparities Research Medical School University of Minnesota Minneapolis, MN United States

**Keywords:** hypertension, mobile health technology, health disparities, randomized controlled trial

## Abstract

**Background:**

Suboptimal treatment of hypertension remains a widespread problem, particularly among minorities and socioeconomically disadvantaged groups. We present a health system–based intervention with diverse patient populations using readily available smartphone technology. This intervention is designed to empower patients and create partnerships between patients and their provider team to promote hypertension control.

**Objective:**

The mGlide randomized controlled trial is a National Institutes of Health–funded study, evaluating whether a mobile health (mHealth)-based intervention that is an active partnership between interprofessional health care teams and patients results in better hypertension control rates than a state-of-clinical care comparison.

**Methods:**

We are recruiting 450 participants including stroke survivors and primary care patients with elevated cardiovascular disease risk from diverse health systems. These systems include an acute stroke service (n=100), an academic medical center (n=150), and community medical centers including Federally Qualified Health Centers serving low-income and minority (Latino, Hmong, African American, Somali) patients (n=200). The primary aim tests the clinical effectiveness of the 6-month mHealth intervention versus standard of care. Secondary aims evaluate sustained hypertension control rates at 12 months; describe provider experiences of system usability and satisfaction; examine patient experiences, including medication adherence and medication use self-efficacy, self-rated health and quality of life, and adverse event rates; and complete a cost-effectiveness analysis.

**Results:**

To date, we have randomized 107 participants (54 intervention, 53 control).

**Conclusions:**

This study will provide evidence for whether a readily available mHealth care model is better than state-of-clinical care for bridging the guideline-to-practice gap in hypertension treatment in health systems serving diverse patient populations.

**Trial Registration:**

Clinicaltrials.gov NCT03612271; https://clinicaltrials.gov/ct2/show/NCT03612271

**International Registered Report Identifier (IRRID):**

DERR1-10.2196/25424

## Introduction

Hypertension (HTN), a major risk factor for strokes and heart attacks, is also a significant comorbidity in severe COVID-19 infections [1-3]. Unfortunately, of the estimated 86 million US adults with HTN, 46% (~40 million) have poorly controlled or uncontrolled HTN [4]. Despite widespread recognition of the health risks of HTN, suboptimal treatment of HTN remains a pernicious problem and is marked by disparities [4,5]. Rates of HTN control are worse among racial and ethnic minorities and socioeconomically disadvantaged groups, who experience a disproportionate burden of cardiovascular disease (CVD) and poor health outcomes [4,5]. According to the 2017-2018 National Health and Nutrition Exam Survey, rates of controlled HTN in Hispanic adults (36.8%) were substantially lower than in non-Hispanic white adults (45.2%) [6]. HTN is undertreated even among stroke survivors who are at significantly increased risk of recurrent stroke [7]. Many factors have been associated with suboptimal HTN control including gaps in health services, lower socioeconomic status, and limited self-care [8-10].

Self-measured blood pressure monitoring (SMBP), an aspect of self-care, is effective in lowering blood pressure (BP) and improving HTN control [11]. SMBP is recommended in guidelines on the care of patients with HTN including the Eighth Joint National Committee [1] and the American College of Cardiology [12,13]. It was also endorsed in a joint policy statement by the American Heart Association (AHA) and American Medical Association as a result of increasing utilization of telehealth visits in the COVID-19 pandemic [14].

Mobile health (mHealth) technology has emerged as an innovative way to facilitate SMBP [15,16]. Our prior pilot study with stroke survivors found that SMBP utilizing mHealth improved rates of HTN control [17]. In a randomized controlled trial (RCT), we compared usual care versus an mHealth-based model of HTN care that included automated wireless transmission of BP data to the provider team, including a clinical pharmacist, who could make responsive medication adjustments. The mHealth care model was feasible and acceptable to stroke survivors and was highly effective: 89% of participants in the mHealth group versus 58% in the usual care group (*P*=.015) had their BP controlled at 3 months postrandomization. However, the generalizability of our results was limited as all the participants were stroke survivors and the majority of participants were English-speaking Caucasians, which is typical of many published studies [15]. To the best of our knowledge, there are no published studies that evaluate mHealth-based HTN care in Hmong patients.

To address these gaps, we designed an RCT, called mGlide, to evaluate whether an mHealth-based active partnership between health care teams and patients results in better HTN control than a state-of-clinical care comparison (usual care) for stroke survivors and persons at elevated risk of CVD. We are particularly interested in addressing disparities in HTN control in vulnerable patient populations in our region. Thus, our recruitment sites include federally qualified health centers (FQHCs) that predominantly serve low-income racial or ethnic minorities and immigrants, including lower socioeconomic groups and African American, Hmong, Somali, and Latino patients. The primary study aim tests the clinical effectiveness of the 6-month intervention vs usual clinical care. Secondary aims evaluate sustained HTN control rates at 12 months; describe provider experiences of system usability and satisfaction; examine patient experiences, including medication adherence and medication use self-efficacy, self-rated health and quality of life, and adverse event rates; and complete a cost-effectiveness analysis. The mGlide RCT began in late 2018, with recruitment initiated in March 2019. Our purpose in this article is to describe the design and rationale of the mGlide RCT study.

## Methods

### Study Design

mGlide is a National Institutes of Health–funded, investigator-initiated, 12-month, 2-arm RCT evaluating HTN control rates between the study intervention and clinical comparison groups. We use a PROBE (Prospective Randomized Open Blinded End-point) design. We are recruiting a total of 450 patients with uncontrolled hypertension who are either stroke survivors or primary care patients at elevated risk of CVD from the metropolitan area of Minneapolis and Saint Paul, Minnesota. The recruitment window is between March 2019 and December 2022. Individual participants are randomized to either the multilevel mGlide intervention (target n=225) or to state-of-clinical care (target n=225) for a 6-month intervention period, followed by a 6-month observation period. A baseline BP assessment and 2 follow-up BP assessments (at 6 months and 12 months postrandomization) are completed for each participant.

**Figure 1:**
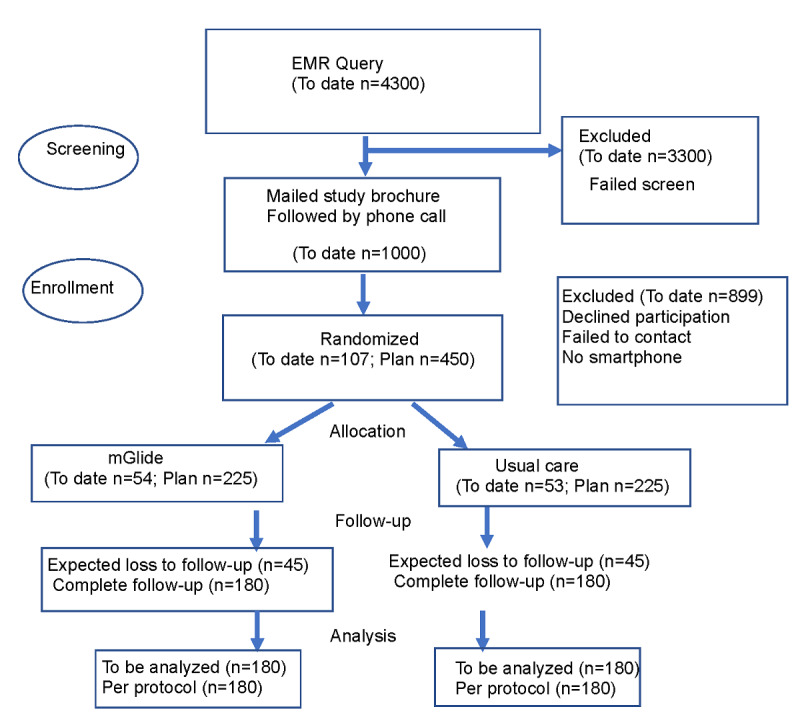
mGlide CONSORT diagram. EMR: electronic medical record.

Eligibility criteria are shown in Textbox 1 and in the CONSORT flow diagram (Figure 1). The University of Minnesota Institutional Review Board (IRB) approved the study protocol, and all participants provide written, informed consent.

**mGlide randomized controlled trial eligibility criteria.**

**Inclusion criteria**

- Aged 18-85 years
- Established medical diagnosis of hypertension (HTN)
- Uncontrolled HTN during screening defined as:
  Systolic blood pressure (SBP) >140 mm Hg at last 2 clinic visits in the 6 months prior to the screening date or
  If a patient is discharged from the hospital in the 6 months prior to screening and does not have 2 clinic visits after hospital discharge, at least 1 SBP in last 2 hospital days >140 mm Hg or
  If only 1 office visit and no hospitalization in last 6 months, then a single SBP in the system >150 mm Hg and SBP >140 mm Hg at an invited pre-enrollment screening visit
- Be at high cardiovascular disease (CVD) or stroke risk, defined as:
  History of ischemic stroke or intraparenchymal hemorrhage or
  History of established CVD disease (coronary artery disease, peripheral vascular disease) or
  Elevated risk of stroke or CVD events as defined by the American Heart Association (AHA)/American College of Cardiology (ACC) guideline on risk stratification ≥7.5% over 10 years if ≥40 years old and ≥10% nonpooled risk calculation if <40 years old
- English, Spanish, Hmong, or Somali speaking
- Have a smartphone or mobile device (eg, iPad) that can transmit blood pressure (BP) from the BP monitor; iOS and Android compatible (iOS 7 or higher: iPhone 4 or higher, iPod touch 5th generation or higher, iPad 2nd generation or higher; Android 4.0 or higher)
- Capable and willing to comply with the entire study protocol
- Able to give voluntary written informed consent

**Exclusion criteria**

- Severe comorbid illness including end-stage kidney disease, end-stage liver disease, and life expectancy <1 year, or if medical complexity of the patient precludes clinical trial participation
- Active illicit drug use (eg, cocaine, methamphetamines, opioids, phencyclidine)
- Unable to complete study tasks, including are homeless, will leave the country, or will relocate in the next 12 months
- Serious psychiatric illness that could interfere with treatment, assessment, or compliance including significant delusional disorders such as schizophrenia and bipolar illness
- Unable or unwilling to give consent

### Study Setting and Recruitment

The study is conducted by the University of Minnesota, a large urban university in the upper Midwest in the United States. Study participants are community-dwelling residents within our 7-county metropolitan area recruited from (1) a large academic health system (Fairview Health System) with a stroke service and primary care clinics; (2) university-affiliated, community-based, primary care clinics serving low-income participants and minorities (University of Minnesota Physicians clinics); and (3) 2 FQHCs: Minnesota Community Care, the largest FQHC in Minnesota, and Neighborhood Health Source, serving low-income people from ethnically diverse communities. Details are in Table 1.

**Table 1:** Planned enrollment (N=450).

Site	Number of participants	Description of recruited participants
Fairview stroke service	100	Stroke survivors
Fairview primary care clinics	150	Elevated CVD^a^ risk
UMP^b^ primary care clinics	125	Elevated CVD risk; low income; diverse; minority
FQHC^c^ clinics (MnCC^d^, NHS^e^)	75	Elevated CVD risk; low income; diverse; minority

^a^CVD: cardiovascular disease.

^b^UMP: University of Minnesota Physicians.

^c^FQHC: federally qualified health centers.

^d^MnCC: Minnesota Community Care.

^e^NHS: Neighborhood Health Source.

Eligible stroke survivors are identified from the Fairview acute stroke service and the acute rehabilitation unit. Primary care patients are identified by electronic medical record queries using the inclusion and exclusion criteria in Textbox 1. Patients who have “opted out” of research are not included in the electronic medical record query (~5% of all patients). Eligible participants are mailed a study brochure and are subsequently contacted by phone. Those who express interest undergo a second screening for availability of a smartphone or mobile device and are invited for a baseline enrollment visit.

### Visit Schedule and Assessments

Each participant completes a total of 3 visits: baseline visit and 2 follow-up visits at 6 months and 12 months postrandomization. The baseline visit includes informed consent in the participant’s language, randomization into one of the 2 study arms, participant education about HTN and BP control, baseline BP measurement using a protocol [18,19], and baseline surveys. The 2 follow-up visits include assessments of the primary and secondary study outcomes. In addition, team members call participants monthly (months 1-5) and bimonthly (months 8 and 10) to identify adverse events and address any challenges. Data collection details are presented in Table 2.

**Table 2:** mGlide randomized controlled trial visit schedule and assessments at baseline and follow-up.

Measures		Baseline	6-month follow-up	12-month follow-up
Demographic information		x	N/A^a^	N/A
Medical history		x	N/A	N/A
Health behaviors (smoking, physical activity, diet, sleep)		x	N/A	N/A
Health insurance		x	N/A	N/A
Blood pressure		x	x	x
**Patient-reported outcomes**				
	Health care experiences (CAHPS^b^ adult survey)	x	x	x
	Hill-Bone Medication Adherence Scale (HB-MAS)	x	x	x
	Medication and Self-Efficacy Scale - Revised (MASES-R)	x	x	x
	Patient Activation Measure (PAM-10)	x	x	x
	Health care utilization	x	x	x
	Self-reported health status (EQ-5D-3L)	x	x	x
	Technology usability (Marshfield System Usability Survey)	N/A	x	N/A

^a^N/A: Not applicable.

^b^CAHPS: Consumer Assessment of Healthcare Providers and Systems.

### Special Considerations for Recruitment

University of Minnesota researchers and community researchers from Hmong and Latino communities co-developed the informed consent and surveys in English. Subsequently, community researchers translated all participant-facing documents into Hmong, Spanish, and Somali. Each participant invited to a baseline enrollment visit works with a team member who speaks their language (Hmong, Spanish, Somali, or English).

### Randomization

Eligible and consented participants are randomized 1:1 to either an intervention mGlide arm or a state-of-clinical-care comparison arm. The randomization uses a site-specific randomization schedule and is stratified across the 4 participant groups discussed in Table 1. Randomization schedules follow from permuted blocks with randomly varying sizes to ensure approximate balance between the 2 study arms in each stratum across the trial while reducing predictability of the assignments.

### Interventions

#### mGlide

The mGlide intervention has 3 components (Table 3): participant education on the importance of HTN control, training on SMBP and wireless transmission of BP, and responsive antihypertensive medication adjustment by the pharmacist-provider team.

Each participant receives education on the importance of HTN control via an IRB-approved educational video available in English, Spanish, and Hmong. We previously developed this video through a community-engagement process led by community researchers from the Hmong and Latino communities. The study team member then trains the participant on SMBP [18] using a wireless BP monitor provided by the study and a smartphone (participant’s own phone or mobile device such as iPad). The intervention participants are requested to self-monitor their BP daily with specific guidance on timing and proper technique based on the AHA recommendations [18]. The self-monitored BP is automatically transmitted to a provider REDCap database and is used for responsive antihypertensive medication adjustment. The participant BP transmission is automated and facilitated by the participant’s mobile device via an app. Pharmacist teams at each clinic location access the BP data via a web-based user interface that identifies patients whose readings were out of bounds during the prior week. This user interface creates efficiencies for pharmacists reviewing the data and was developed to avoid information overload for pharmacists. Pharmacists adjust medications based on collaborative practice agreements with primary care providers (PCP) and mGlide protocols. Pharmacists adjust medications and communicate with the patient and their PCP as often as every 2 weeks or as needed to reach the BP goal.

**Table 3:** Components of mGlide intervention delivered to participants.

Component	Intervention	Comparison
Education on importance of hypertension control	Yes	Yes
Training in blood pressure self-monitoring	Yes	Yes
Daily self-monitoring	Requested	Encouraged
Automated wireless blood pressure transmission via mobile device	Yes	No
Responsive medication adjustment by pharmacy	Yes	No
Primary care follow-up as usual	Yes	Yes

Our intervention is a multilevel model with a 3-member team: frontline research team member who educates the participant and trains them on self-monitoring of BP, pharmacist who provides responsive HTN management, and PCP. This team communicates via the electronic medical record as part of clinical care.

#### State-of-Clinical Care Comparison

Each study participant randomized to the clinical care comparison group receives the same education as intervention participants on the importance of HTN control via the IRB-approved educational video. The comparison group participants receive a digital BP monitor and are trained on BP self-monitoring with proper technique. Participants are encouraged to measure their BP daily and follow up with their PCP regarding their BP, as requested by the PCP as part of their clinical care. Participants, PCPs, and clinic teams are responsible for communicating as they would in usual clinical care. In other words, there is no predetermined follow-up schedule with the PCP specified by the study, and there is no automatic transmission of participant BP readings to providers; rather, participants are responsible for sharing their BP and working with their care team to manage their BP.

### Analysis

#### Outcomes

The main outcome will be the rate of HTN control at 6 months; the primary outcome will be defined as a binary indicator of uncontrolled HTN or death versus controlled HTN at 6 months. The BP is measured in-person in the clinical research center by a trained staff member who is blind to the patient’s assignment. The BP is measured 4 times using a standard digital monitor using proper technique, and the average of the last 3 BP measurements denotes the BP outcome. HTN control is defined as systolic blood pressure (SBP) <140 mm Hg, which is consistent with American College of Physicians, American Association of Family Physicians, and AHA primary and secondary stroke prevention guidelines [12,13,20]. The 2017 American College of Cardiology/AHA guidelines recommend a SBP goal of <130 mm Hg. The HTN treatment goals and target BP were discussed with the PCPs and clinical pharmacists at all sites prior to study recruitment. Everyone agreed to target a SBP goal of <140 mm Hg for intervention arm participants and requested that lower SBP goals be left to the discretion of the study pharmacists managing the patient. Study pharmacists have access to patient medical records. At 12 months, the BP outcome is assessed in the same manner as at 6 months. This allows the assessment of the secondary outcome of uncontrolled HTN or death versus controlled HTN at 12 months. Additional secondary outcomes include patient-reported medication adherence, medication use self-efficacy, self-reported health status, and quality of life that we assess with validated questionnaires at each of the 3 study visits. We ascertain participant adverse events and health care utilization during monthly phone calls during the first 6 months of the study and bimonthly in months 7-11. We also assess care providers’ experience of the mGlide system usability.

#### Power and Sample Size

Sample size was calculated to address outcomes of failure to achieve HTN control or death at 6 months and 12 months after randomization. We based sample size calculations on (1) reported 1-year stroke survivor mortality rate of 4% [21] and assuming similar mortality among primary care participants with elevated CVD risk, (2) pilot results that showed a primary outcome rate of HTN control or death at 6 months of 15% in the intervention and 50% in the comparison groups [17], and (3) a 20% attrition rate. We identified that a sample size of 450 would provide at least 85% power, with an alpha of .05 to detect a 15% effect size. We planned for a more modest intervention effect size than achieved in our pilot results due to the longer period of observation and more diverse health systems and patient populations when compared to the pilot study. Similarly, while the loss to follow-up in the pilot study was very modest at 4%, we planned for a 20% attrition rate due to the economically stressed primary care population in our low-resource health systems. Nationally reported rates of uncontrolled HTN in stroke survivors are ~50% [7]. FQHC primary care rates of uncontrolled HTN are ~38%-40% [22]. Our sample size will allow valid subgroup analysis among the subgroup of primary care patients in all low-income sites (n=200) and the stroke survivor subgroup (n=100).

#### Quality Control and Preventing Missing Data

Data collection is standardized by the use of a detailed manual of operations. The REDCap data entry system also has built-in logic to check data at the time of entry and minimize errors. Monthly data quality reports ensure that data are validated and data entry is completed in a timely fashion. If participants drop out, we are documenting specific reasons for drop out.

#### Planned Analysis

We will use a Cochran-Mantel-Haenszel test stratified by the randomization groups to test whether the odds of HTN control at 6 months differs between the intervention and control study arms. Statistical significance level will be set at the *P*<.05 level. We also will perform the same analysis at 12 months to examine if the difference between the intervention and control groups is sustained. Secondary analyses using the repeated BP assessments obtained at 6-month and 12-month visits are planned. We will use longitudinal generalized estimating equation models, with BP control modeled as a binary variable and average BP modeled continuously in separate models, with relevant covariates. Interactions will be tested to evaluate potential differential effects by baseline demographic and clinical characteristics.

We have planned for missing data. We are recording reasons for study drop out and will examine patterns of missingness. For the primary analysis, we will (multiply) impute the binary HTN control outcome. We are collecting a rich amount of covariate information on participants to inform the multiple imputation models.

We plan the following analyses to examine secondary outcomes related to system usability for care providers. The Welch *t* test will be used to examine the number of antihypertensive medication changes (dose adjustment, addition of new medications) per patient during the 6-month intervention period for the mGlide intervention arm. We will examine the provider mGlide experience using the Computer System Usability Questionnaire overall score as well as scores along the 3 principal factors identified on the Computer System Usability Questionnaire: System Usefulness, Information Quality, and Interface Quality. We also will collect qualitative feedback from providers using focus groups. These data will be analyzed using a qualitative framework including classic content analysis and microinterlocutor analysis [23,24].

Patient-reported outcomes (Table 2) will be examined by comparing whether there are group differences on the Patient Activation Measure [25], patient medication self-efficacy measure (Medication and Self-Efficacy Scale - Revised) [26], medication adherence (Hill-Bone Medication Adherence Scale) [27,28], self-reported health status (EQ-5D-3L) [29,30], and patient satisfaction with health systems and providers (Consumer Assessment of Healthcare Providers and Systems adult survey) [31] using a 2-sample *t* test separately at 6 months and 12 months after baseline. Finally, we will compare the rates of adverse events between the 2 trial arms using both adjusted and unadjusted negative binomial regression models with intervention as the key independent variable.

#### Additional Analyses

We plan a cost-effectiveness analysis that will follow the recommendations of Cost-Effectiveness in Health and Medicine, 2nd Panel [32] to present the analysis from both the societal and health care perspectives and to include an inventory of the non-health care impacts of the mGlide intervention. We will measure effectiveness by the differences in quality-adjusted life years (as derived from the EQ-5D-3L quality-of-life weights) across the 2 trial arms. Costs will represent the intervention cost and any differences in downstream health care and other costs for the societal perspective (productivity or time, informal care, travel) across the 2 arms. We will extrapolate quality-adjusted life years and cost differences using a 10-year stochastic event model. Thus, the cost-effectiveness analysis will meld both the trial follow-up experience of participants and their modeled experience for the remaining years, based on differences in HTN control rates between the 2 trial arms. Specifically, we will determine downstream health care utilization using a state transition (Markov) model using Monte Carlo microsimulation. Our team includes an experienced health economist (JN).

### Data Safety and Monitoring

We have convened a 5-member data safety monitoring board (DSMB) with expertise spanning the statistics of clinical trial monitoring, HTN, pharmacist-delivered care, vascular neurology, and cardiology. Following an initial meeting at study start, the DSMB meets approximately every 6 months.

## Results

Study enrollment commenced in March 2019. Through mid-March 2020, 101 participants were randomized with 52 to the mGlide arm and 49 to the state-of-clinical-care arm. In mid-March 2020, the University of Minnesota paused enrollments for non-COVID-19 research studies involving face-to-face participant contact. In response, the mGlide team developed protocols for remote enrollment including remote consent in REDCap and protocols to mirror the in-person enrollment process. We also developed a remote protocol for gathering the 6-month and 12-month follow-up data and BP measurements using Zoom. These have been approved by the IRB and the study sponsor and are currently in use. To date, we have enrolled 5 participants remotely and are completing the 6-month and 12-month follow-up visits. We will validate our remote BP measurement protocols against pre-COVID-19 in-person processes when our institution allows in-person research participant visits. The impact of COVID-19 is that the study timeline for completion of enrollment and intervention has been extended by 6 months; other possible effects on the study will be monitored.

To date, we have enrolled a total of 107 participants (54 intervention and 53 control participants). A total of 86 participants have completed their 6-month follow-up (primary BP endpoint), and 46 participants have completed their 12-month follow-up (secondary BP endpoint). Seven participants have withdrawn, and 1 participant has died. The study principal investigator reviews adverse events, and a team of 2 blinded study clinicians subsequently reviews these events. The study statisticians and DSMB review these and other study outcomes at the DSMB meetings.

## Discussion

HTN is a chronic disease requiring sustained efforts for long-term control. The mGlide RCT seeks to address the persistent and prevalent clinical challenge of poorly controlled HTN in a diverse sample of adults at elevated risk for stroke and CVD events. The study aims are to (1) evaluate clinical effectiveness of the mGlide intervention in comparison to usual clinical care; (2) improve clinical teams’ abilities to manage patients’ antihypertensive medications; (3) increase patient activation, patients’ satisfaction with care, and medication use self-efficacy and adherence as well as lower health care utilization; and 4) establish the cost-effectiveness of the mGlide intervention. Intervention participants monitor their BP daily with a wireless BP monitor and use their smartphone to transmit BP readings to a database automatically via an app. We then use the framework of glide paths to manage the transmitted BP data. The name of the intervention, mGlide, derives from the glide path concept of landing an airplane; an expected trajectory of BP readings is established for each patient with bounds set by guidelines and further adjusted by providers as needed. Although BP is monitored daily at home, the health care team accesses the BPs once a week and makes medication adjustments as needed, in collaboration with the patient’s PCP. We believe this approach will facilitate early intervention in an efficient manner while avoiding system information overload.

The key innovation of the mGlide trial is using a mobile technology platform to facilitate better HTN control through a collaborative patient-provider partnership in limited-resource health systems. Currently, 68% of US adults use smartphones (up from 35% in 2011) [33]. The mGlide system based on mobile technology uses the patient’s own smartphone. A free app allows the wireless monitor to interface with the smartphone. The app transmits the data to an online database. The database is free, and there is no patient service contract. Hence, mHealth is nimble and represents the next generation in technology. While small clinical trials have demonstrated the efficacy of mHealth in SMBP [11,15-17], we will demonstrate the feasibility of the mGlide in different clinical health systems including low-resource environments that might not be able to afford investment in telemonitoring services with an outside vendor.

Results from this study will provide evidence for the use of readily available mHealth technology for bridging the guideline-to-practice gap in HTN treatment for diverse patients in diverse health care systems. Importantly, our study is being implemented in low-resource health systems serving minority and low-income groups and thus will provide critical insights into enhancing HTN control in these elevated-risk patients who experience significant cardiovascular disparities.

## Appendix

Multimedia Appendix 1 Peer-review reports from the NIH.

## Abbreviations

AHA: American Heart Association
BP: blood pressure
CVD: cardiovascular disease
DSMB: data safety monitoring board
FQHC: federally qualified health centers
HTN: hypertension
IRB: institutional review board
mHealth: mobile health
PCP: primary care provider
PROBE: Prospective Randomized Open Blinded End-point
RCT: randomized controlled trial
SBP: systolic blood pressure
SMBP: self-measured blood pressure monitoring
