# Supplementary material for: Mobile Health Intervention to Close the Guidelines-To-Practice Gap in Hypertension Treatment: Protocol for the mGlide Randomized Controlled Trial
Source: JMIR Res Protoc. 2021 Jan 25;10(1):e25424. doi: 10.2196/25424 (PMC7870345; doi:10.2196/25424)
Supplement: Multimedia Appendix 1 [file resprot_v10i1e25424_app1.pdf]

SUMMARY STATEMENT
(Privileged Communication)

PROGRAM CONTACT:
CATHERINE STONEY
301-435-6670
stoneyc@mail.nih.gov

Release Date: 11/08/2017
Revised Date:

Application Number: 1 R01 HL138332-01A1

Principal Investigator

LAKSHMINARAYAN, KAMAKSHI

Applicant Organization: UNIVERSITY OF MINNESOTA

Review Group: ZRG1 HDM-G (59)
Center for Scientific Review Special Emphasis Panel
PAR 15-279: Strategies to Increase Delivery of Guideline-Based Care to Populations with Health Disparities

Meeting Date: 11/02/2017
Council: JAN 2018
Requested Start: 03/01/2018
RFA/PA: PAR15-279
PCC: HHCG N

Project Title: mGlide RCT: A Clinical Glide Path to Close the Guideline-to-Practice Gap in HTN Management
SRG Action: Impact Score:33 Percentile:19 #
Next Steps: Visit https://grants.nih.gov/grants/next_steps.htm
Human Subjects: 30-Human subjects involved - Certified, no SRG concerns
Animal Subjects: 10-No live vertebrate animals involved for competing appl.
Gender: 1A-Both genders, scientifically acceptable
Minority: 2A-Only minorities, scientifically acceptable
Children: 3A-No children included, scientifically acceptable
Clinical Research - not NIH-defined Phase III Trial

Project Year	Direct Costs Requested	Estimated Total Cost
1	612,448	884,939
2	542,117	783,316
3	549,584	794,106
4	566,965	819,220
5	564,259	815,310
TOTAL	2,835,373	4,096,891

EARLY STAGE INVESTIGATOR
NEW INVESTIGATOR

1R01HL138332-01A1 LAKSHMINARAYAN, KAMAKSHI

EARLY STAGE INVESTIGATOR
NEW INVESTIGATOR

RESUME AND SUMMARY OF DISCUSSION: This application proposes to use a randomized control trial design to evaluate the effectiveness, along with other implementation components of mGlide, a mobile health technology that engages patients with uncontrolled hypertension (HTN) with their physicians to co-manage their blood pressure. If successful, this study will provide evidence supporting mHealth technology for the management of HTN among minority patients in low-resource areas. During the discussion, the panel agreed that the application was highly significant and the scientific premise was sound as evidenced by the disproportionately higher rates of uncontrolled HTN among minority populations compared to Whites in the United States and preliminary data results demonstrating the acceptability, feasibility and efficacy of implementing the mGlide intervention for controlling HTN among stroke survivors. The reviewers agreed that the investigative team had impressive backgrounds, history of collaboration and a strong supportive environment to achieve the study's aims. The panel viewed the idea of an mHealth intervention that wirelessly monitors blood pressure, as somewhat innovative. The committee agreed that the resubmission was adequately responsive to previous critiques. The panel identified additional strengths, including: strong involvement from stakeholders; appropriately described multi-level approach; adequate power calculations; and a strong data analysis plan that addressed sex as a biological variable. The committee also noted weaknesses, including: concern about mGlide operating outside of the electronic medical record; lack of clarity about where the mGlide data will be housed and who will have access to it; and insufficient details regarding access to and recruitment of minority patients, weakening the scientific rigor. Reviewers viewed these weaknesses as easily addressable and did not detract from the application's high merit. Overall, the panel expressed high enthusiasm for the application and agreed that the study's outcomes would have a high impact on using mHealth technology to prevent stroke and improve cardiovascular health in low resource health systems serving minority groups.

DESCRIPTION (provided by applicant): This A1 application is in response to PAR-15-279, "Strategies to Increase Delivery of Guideline- Based Care to Populations with Health Disparities." Hypertension (HTN) is the most important stroke and cardiovascular disease (CVD) risk factor. Unfortunately, there is substantial under-treatment of HTN. Of the 86 million adults with prevalent HTN in the U.S., 40 million (46%) have inadequately controlled blood pressure (BP). This problem is worse among minority groups. In this study, we demonstrate how mHealth (mobile health technology) can improve HTN control rates in stroke survivors and primary care patients without stroke, but who are at a high risk of stroke and CVD. Our intervention is called mGlide. Intervention participants will self- monitor their BP daily using a wireless BP monitor and a smart phone. The phone will transmit this BP to a database automatically. We will use the framework of glide paths to manage the transmitted BP data. The glide path, based on the concept of landing an airplane, establishes an expected trajectory of BP readings for each patient with bounds set by guidelines and provider input. BP is monitored at home; the health care team is alerted when patient BP deviates from expected bounds. Alerts are generated once a week for the health care team with a list of patients with uncontrolled HTN. This facilitates early intervention while avoiding information overload. In a pilot study (R21HS021794), we randomized 50 stroke survivors to mGlide (n=26) vs. usual care (n=24). Our intervention team included a front-line coordinator who trained the patient on BP self-monitoring and a pharmacist who co-managed the patient's HTN with the patient's physician. Our results confirm excellent acceptability, high feasibility and promising efficacy of mGlide for achieving HTN control. Based upon this 4- month pilot we propose a longer trial. Our partnering clinical centers include Federally Qualified Health Centers that serve low income and minority (Latino, African American, Hmong) communities. In this RCT study, we will randomize 450 participants with uncontrolled HTN to the mGlide intervention (n=225) vs. state-of-clinical-care comparison (n=225). Aim 1 will examine how well HTN is controlled in the two groups at 6

months and 12 months after randomization. Aim 2 will examine mGlide usability for providers and provider experience and satisfaction with mGlide. It will also examine whether medications are managed differently for participants in the two groups. Aim 3 will examine whether patients are more satisfied with care in the mGlide group, whether they are more “activated” and have a greater sense of self-efficacy in managing their HTN. Aim 4 will be a cost-effectiveness analysis of providing mGlide care. Our long-term goal is to prevent stroke and improve cardiovascular health in populations by increasing health system efficiency and effectiveness. The results from our mGlide RCT will provide evidence for the use of readily available mHealth technology for bridging the guideline-to-practice gap in HTN treatment in low resource health systems serving minority groups.

PUBLIC HEALTH RELEVANCE: This study addresses the significant public health problem of uncontrolled hypertension and can prevent stroke and improve cardiovascular health while reducing costs. The current U.S. annual estimated cost of hypertension is $51 billion. Results from this study will provide evidence for the use of readily available mobile health technology for bridging the guideline-to-practice gap in hypertension treatment. Our study will be implemented in low resource health systems serving minority groups. We also include an economic analysis showing the cost-effectiveness of our mobile health technology based care model.

CRITIQUE 1

Significance: 3
Investigator(s): 1
Innovation: 2
Approach: 2
Environment: 1

Overall Impact: Control of hypertension is important for a number of reasons but remains suboptimal. This is a 12-month RCT (6-month active intervention) using mHealth wireless Self Monitored BP measurements fed into a semi-automated decision support tool (called mGlide) compared to state of clinical care targeting 3 minority groups with low rates of hypertension control. The primary outcome is “rate of HTN control”. The work is grounded in the premise that HTN control can be improved if one can affect two changes: 1) get people to self-monitor BP (SMBP) and 2) get clinicians to closely and actively adjust medications. While the causes of the “guideline to practice” gap in HTN control are undoubtedly complex their premise is supported by high quality evidence. The approach used has high scientific rigor and use of wireless monitoring is innovative as is use of mGlide. A successful outcome (positive result) would be highly significant in that an approach to improved HTN control in one or several of their high-risk minority groups would provide generalizable guidance for others to follow in similar populations. Sex as a bio variable was adequately addressed. The resubmission addresses the major concerns of the first review. Overall the study is considered likely to have high impact.

1. Significance:

Strengths

- The premise is supported by prior work cited in the literature.
- Their preliminary results from a 50-person short term study indicate that it is likely that patients will use the monitor and report data (at least for a while), a key point as digital home monitors have been around for some time but patient use is often irregular. Preliminary data even suggest efficacy although the study was not long term. The underlying premise is that if one can get patients to reliably stream the measurements an activation and a degree of accountability are created---and under such conditions it is likely patient compliance with meds will be good.

- Successful demonstration of improved control with this approach would be very significant as smart phones are very prevalent and the decision support approach can be scaled and translated to health systems of varying sizes and configurations.

Weaknesses

- Minor: data supporting the idea that the flow of BP information will be both regular and sustained are limited (89% of 120 days with no information on trend).
- Minor: Data on physician acceptance and smoothness of workflow integration are still being collected.
- Minor. There is no justification for the assumption that the mHealth and wireless monitor will be used regularly when portable digital monitors do not seem to be. Also, it is less clear how important physician management (one of the outcomes is how often changes in meds are made---Is it the meds or the fact that patients don't take the meds?).

2. Investigator(s):

Strengths

- The full complement of investigators needed to carry out this work are in place including: focused PI who, despite being a New Investigator, has completed the preparatory pathway (K23) toward independence in this sort of research and has experienced guidance (e.g. Russell Luepker and many others); experienced clinical trials biostatistician (John Connett); clinical community (including minority experience) based co investigators/trialists (Kathleen Culhane-Pera and Shannon Pergament); cost effectiveness analysis (John Nyman); Pharmacy (Sarah Marie Westberg); large trials involving hypertension (Russell Leupker) and others.

Weaknesses

- None noted.

3. Innovation:

Strengths

- mHealth and wireless BP monitor.
- mGlide decision support pathway.

Weaknesses

- Minor: The lack of significant participation of the health care system which seems to play the role of allowing the research to go forward rather than finding novel ways to support (see Approach) it is not novel.

4. Approach:

Strengths

- The individualized RCT approach is seen as a strength.
- The evaluation of intervention impact on providers, patients and costs is seen as a strength.
- The comparison arm is well described.

- Approach is seen as properly navigating the tension between having a “multilevel” approach and keeping the intervention focused and finite enough so that conclusions can be drawn from the results.

Weaknesses

- Minor: The design lacks incentives for the physicians.

5. Environment:

Strengths

- This is a very strong environment with great experience in epidemiology, public health and clinical trials---and a community based practice network with appropriate access to and experience with the target minority groups.

Weaknesses

- The previously noted deficiencies in the Protection of Human Subjects and DSMB configuration have been addressed and the age inclusion range widened.

Protections for Human Subjects:

Acceptable Risks and/or Adequate Protections

Data and Safety Monitoring Plan (Applicable for Clinical Trials Only):

Acceptable

Inclusion of Women, Minorities and Children:

- Sex/Gender: Distribution justified scientifically
- Race/Ethnicity:
- For NIH-Defined Phase III trials, Plans for valid design and analysis: Scientifically acceptable
- Inclusion/Exclusion of Children under 18: Including ages <18; justified scientifically

Vertebrate Animals:

Not Applicable (No Vertebrate Animals)

Biohazards:

Not Applicable (No Biohazards)

Resubmission:

- Adequately addressed the prior critiques.

Applications from Foreign Organizations:

Not Applicable (No Foreign Organizations)

Select Agents:

Not Applicable (No Select Agents)

Resource Sharing Plans:

Acceptable

Authentication of Key Biological and/or Chemical Resources:

Not Applicable (No Relevant Resources)

Budget and Period of Support:

Recommend as Requested

CRITIQUE 2

Significance: 6
Investigator(s): 4
Innovation: 7
Approach: 6
Environment: 2

Overall Impact: This proposal focuses on automated home blood pressure monitoring to improve hypertension control and reduce the risk of stroke. Hypertension and increased risk of stroke are clearly very common and important health concerns and fit well within FOA's specified subjects for consideration. Both the PI and Co PIs are well trained and published in this area. A pilot study apparently has been completed, but is inadequately presented in this application. Data important to the success of the study is often cited as references to the literature or to even biosketches, instead of a one or two sentence summary to support the concept being discussed. This raises concerns whether there is adequate focus and organizational capacity to effectively implement a complex study regarding the use of automated, home blood pressure monitoring to achieve normal blood pressures in hypertensive patients. This reviewer also has significant concerns about recruitment of patients from the Fairview Health System, which is not well described in the protocol. The Fairview web page describes six hospitals, 39 primary care clinics, 34 pharmacy locations, and 4000 doctors and providers in 30 cities across Minnesota. If typical of university affiliated health care systems, it is unlikely that Fairview has a focus on health disparities or economically distressed patients, as opposed to the two FQHCs included in the protocol. This reviewer was unable to find criteria in the protocol for patient recruitment that focuses on health disparities within the Fairview system. Data from table 8 show that 93% (22,033/23,687) of the "high risk" (not defined in the protocol) patients with uncontrolled hypertension are available in the Fairview system, just 7% in the FQHCs; this very large difference is not credible. Although recruitment will be limited to 11 Fairview clinics, there is no discussion of focusing on health disparities. Both the control and intervention arms will do home BP monitoring – although using different monitoring systems with electronic transmission back to the research team occurring only in the intervention arm, which includes a pharmacist. Engagement of the primary care provider appears to be similar in the two arms. It appears that the electronic feedback of BPs does not get to the electronic health record and available to non-study care providers, except as recorded in text notes by the study pharmacist.

.

1. Significance:

Strengths

- Hypertension is a common health problem, and better control will reduce the risk stroke.

Weaknesses

- Page 159, Specific Aim 1, MODERATE: The statement, “rates of HTN treated into optimal range in the 2 groups at 6 months and 1 year after randomization”, does not specify which of the many blood pressure measurements available to the study team will be used to assess this comparison.
- Specific Aim 2 is not very specific, MODERATE: a) What is meant by provider actions on anti-HTN medications -- new prescriptions or prescription dose changes? Why are focus groups regarding provider experience included on the same aim?
- Same for Specific Aim 3, MODERATE; At least a dozen comparisons are listed.
- Specific Aim 4, MAJOR: Why are cost-effective analyses (CEA) being done using data from the literature?
- MODERATE, Specific aim 4 states, “CEA will model the experience of the two trial arms over a 10-years” however, Specific Aim 1 says that this is a “12-month, 2-arm RCT”.
- MODERATE, Hypothesis 1.a. At what time point will SBP be < 140? “Hypertension is a chronic disease. Can’t SBP control be monitored and sustained beyond 1 year as stated in this hypothesis? After all this is a four-year study.
- MAJOR, The Impact statement at the bottom of page 159 states, “This study can … prevent stroke ….” This is an incorrect statement; stroke incidence is not being measured.

2. Investigator(s):

Strengths

- The investigative team has published extensively in the area of hypertension control.

Weaknesses

- None noted.

3. Innovation:

Strengths

- Table 2, page 161, nicely summarizes how the present proposal fits the six pillars of the chronic care model (CCM).
- Page 162: The pilot study showing BP control in 89% (23/26) in the intervention group versus 54% (13/24) in the control group.
- Page 161: both arms will have an ambulatory BP monitor and will be educated on self-monitoring.
- Page 163: *5.4.1 Experience with research involving minority population, MODERATE:* The experience of Dr. Culhane-Pera in the Hmong population is an important strength, but not necessarily innovative.

Weaknesses

- Page 161 5.1 MODERATE: The focus group to provide input on mobile device preferences was done five years ago in the Fairview health system, and is not necessarily reflective of the current views of disadvantaged patients.
- Page 161, 4.1: MAJOR: The statement, “The CCM has been effective in health systems but has not been implemented using mobile technology”, is supported by a 2002 publication (reference 42); however, the title of reference 46, published in 2013, includes the phrase, “home blood pressure telemonitoring”.

- Page 162, Lessons learned, MODERATE: No data are presented to support the statement, “The mHealth system was efficacious”.
- Page 162 MILD: Reference 11 cited for the pilot study has no journal or publication name in the reference citation.
- Page 162 MODERATE: The difference between “mhealth facilitated self-monitoring” in the intervention group and “digital monitoring” in the control group is not clear.
- MAJOR: Table 5 presents data that two (WEST SIDE COMMUNITY HEALTH SERVICE and Community University Health Care Center) of the three recruitment health sites focus their care on disadvantaged patients (i.e. are FQHCs), but not Fairview Health Services, which appears to be a consortium of hospitals and clinics owned by or closely affiliated with the University of Minnesota. We are not told how patient selection from Fairview will focus on disadvantaged patients.
- Page 162. 5.3 Formative research toward a mobile technology facilitated chronic care model, MAJOR: The specific aims of this project in the Fairview Health System are presented, but no results are provided. This would be more relevant to the present proposal if were done in one of the FQHCs.
- Page 163, 5.5 Engagement of Stakeholders, MAJOR: Tables 4 and 5 are overly generic and read as engagement to be accomplished, rather than begun. Stakeholder engagement is sufficiently important that a detailed description should be provided in the Research Plan, opposed to referring to letters of support and the protection of human subjects’ section.
- MODERATE: It is not clear why Fairview Health Services clinics have been included as recruitment sites, as they do not appear to have a focus on disadvantaged patients

4.Approach:

Strengths

- None noted.

Weaknesses

- Page 166, 6.5.3: Eligible stroke survivors, MODERATE: Referencing a clinical practice guideline for incidences of hypertension and discharge home among stroke survivors is weak; it would have been much better if these data came directly from the population being considered for study. Also, it would be better if all of these study participants were recruited from the FQHCs, where patients are more likely to meet the definition of having a health disparity, rather than the Fairview Health System.
- Page 166, 6.5.4 Eligible high risk primary care participants: MAJOR: The term, high risk, is used throughout this proposal without defining how the patient values will be obtained. High risk is defined as “> 7.5% CVD risk / 10 years” citing stroke statistics from the American Heart Association (reference 56). However, the data elements required for this risk estimate are not provided; nor are we told how these data will be acquired.
- Page 166, Table 8, MODERATE: The inclusion of 66,768 as “eligible primary care patients at high risk” from the Fairview Health Care system is misleading. We are not given data on the characteristics of the 48 Fairview primary clinics, but one has to presume that they are not FQHCs and represent a heterogeneous group of private care ambulatory facilities caring for patients not insured by Medicaid or otherwise economically disadvantaged.
- Page 166, 6.7.1 Intervention description, MAJOR: Details on remote monitoring of BP are very sketchy; e. g. What is the frequency of the BP measurement? What kind of cuffs are used? How are spurious values dealt with? It would have been much better to include the PI’s description here of the Withings Remote Monitoring System rather than refer to a letter from company that makes the device.

- Page 167, 6.8 Outcomes, key variable description, MODERATE: BP control is the primary outcome; however, we are not told which of the numerous BP recordings are being compared between the two study arms.
- A very large number of additional outcomes are being collected without statements about how they might be used in implementation of the study findings in other care settings.
- Page 167, Table 10 Comments, NINDS CREST2, MODERATE: The cited reference, (52), a web page for the CREST 2 trial, does not describe a protocol for measuring BP. It would have been better to summarize a protocol for measuring BP here: e.g., body position during measurement, sitting quietly with no significant exercise for several minutes, avoidance of isometric muscle contraction, etc. The American Heart Association web page, Monitoring Your Blood Pressure at Home (http://www.heart.org/HEARTORG/Conditions/HighBloodPressure/KnowYourNumbers/Monitoring-Your-Blood-Pressure-at-Home) is an excellent source for this information.
- *Page 167, 6.7.3 Participant contact, MODERATE:* The monthly (first six months) and q 2 month contact with participants in both study arms is not defined. Will the contact be the same for both the intervention and control arms?
- *Page 167-8 Table 10 and Table 10 Comments, MODERATE:* These are very difficult to comprehend; for example, consecutive sentences in the Comments deal with two very disparate concepts: Varimax rotation in principal factor analysis and focus groups on barriers and solutions for translation of mGlide into clinical practice.
- MODERATE: Table 10 and Table 10 comments encompass nearly a full page of the seven pages devoted to 6.0 RESEARCH DESIGN AND METHODS.
- *Page 168,* 6.9 Randomization and blinding. MODERATE: The protocol states that randomization will be “site-specific”, but “site: is not defined. This is important because the number of randomization blocks is dependent on this definition.
- 6.10 Data coordination, management, missing values, quality control MAJOR: This critically important facet of the study is incomplete with only nine lines of text.
- 2) data on provider actions (to be abstracted from EMR), MODERATE: What actions? Prescriptions? Such EMR data abstraction is not a simple task. How will it be standardized? Will inter-observer variability be assessed?
- MAJOR: Although details are not provided, it is difficult to believe that the likely privately insured population of the Fairview system is representative of a population with health disparities.

5. Environment:

Strengths

- The University of Minnesota is very strong in primary care research.

Weaknesses

- None identified.

Protections for Human Subjects:

Acceptable Risks and/or Adequate Protections

- This 10-page Protections for Human Subjects contains much information on the conduct of the clinical trial.

Data and Safety Monitoring Plan (Applicable for Clinical Trials Only):

Acceptable

- The Data Safety Monitoring Plan appropriately covers the following four topics: 1) The Data Safety and Monitoring Board DSMB, 2) Interim Analysis and Stopping rules, 3)

Data Management and Safety, and 4) Adverse event ascertainment, management and reporting.

Inclusion of Women, Minorities and Children:

- Sex/Gender: Distribution justified scientifically
- Race/Ethnicity: Distribution justified scientifically
- For NIH-Defined Phase III trials, Plans for valid design and analysis: Scientifically acceptable
- Inclusion/Exclusion of Children under 18: Excluding ages <18; justified scientifically
- The above are appropriately covered in the Research Plan

Vertebrate Animals:

Not Applicable (No Vertebrate Animals)

Biohazards:

Not Applicable (No Biohazards)

Resubmission:

- This resubmission adequately addresses most concerns of the previous review -- particularly whether this is a population with health disparities.

Applications from Foreign Organizations:

Not Applicable (No Foreign Organizations)

Select Agents:

Not Applicable (No Select Agents)

Resource Sharing Plans:

Not Applicable (No Relevant Resources)

Authentication of Key Biological and/or Chemical Resources:

Not Applicable (No Relevant Resources)

Budget and Period of Support:

Recommended budget modifications or possible overlap identified:

- The budget is essentially flat across all five years of this study; the major expenses will occur during the recruitment and follow-up phases; the budget does not clearly reflect this.

CRITIQUE 3

Significance: 2
Investigator(s): 1
Innovation: 3
Approach: 2
Environment: 1

Overall Impact: This is an excellent application from a new investigator supported by a team of senior researchers and clinicians. Scientific premise and scientific rigor are high and the project will have high impact. It will examine an m-Health intervention for improving guideline-based management of blood pressure implemented in three clinics with three populations experiencing health disparities related to heart disease and stroke prevention – Hmong, African American, and Hispanic patients. Innovation is moderate due to the use of an m-Health intervention and the focus on the Hmong patients. The partnership with the health care organizations is strong and will provide access to the target populations. The investigators improved the design and measures. Analysis methods are strong and will assess clinical and implementation outcomes of the mGlide. Sex as a biological variable is addressed adequately. The remaining weaknesses are judged to be mostly minor with the largest shortcoming being that the trial appear to focus more on the clinical impact on patients than on implementation practices of the providers. The strengths outweigh the minor weaknesses.

1. Significance:

Strengths

- The proposed research seeks to increase adherence to guidelines for treatment of hypertension, which is a risk factor for heart disease and stroke.
- The target populations are Hmong, African American, and Hispanic adults, three populations that experience health disparities.
- Scientific premise for the study is provided by the applicants from the published literature and their preliminary research on feasibility and usability of the m-Glide intervention.
- A relatively simple mHealth intervention, mGlide, will monitor and transmit information on patients' blood pressure to their health care team.
- Effectiveness and cost-effectiveness will be analyzed along with acceptability of implementation with providers and patients.

Weaknesses

- The focus is primarily on the mGlide intervention's effects on patients, with assessment of implementation in the health care system much more limited. (minor)
- Results from the team's ongoing pilot study on provider experience with mGlide (i.e., workflow integration) would strengthen the scientific premise by demonstrating that providers will use mGlide with patients. (minor)

2. Investigator(s):

Strengths

- The PI is a new investigator who has a supportive team of senior researchers and clinicians.
- Team is multi-disciplinary with expertise in stroke and heart disease, biostatistics, pharmacy, health disparities, and cost-effectiveness.
- Site leaders from the three participating health clinics are on the research team.
- The research team has experience recruiting Hmong, Hispanic, and African American patients.

Weaknesses

- None noted.

3. Innovation:

Strengths

- The m-Health intervention implemented in a health care system is somewhat novel.
- The focus on the Hmong population is innovative.
- Involving patients in blood pressure monitoring is unique.

Weaknesses

- Simple reports on blood pressure sent to health care team is not very innovative. (minor)

4. Approach:

Strengths

- A community advisory board will provide input on the research. Stakeholders will be engaged from the patient group, community partners, health care system, and state department of health.
- The three participating clinics will provide access to a sufficient number of Hmong, African American, and Hispanic patients to achieve the planned sample. Plans for recruiting and retaining patients are adequate.
- The intervention is somewhat multi-level, with most of the contact with patients through the mGlide and integration into the health care system via reports to providers.
- The mGlide intervention and measures in the trial are guided by the PRISM framework for intervention implementation.
- The investigators considered and rejected group randomization. They will implement measures of contamination.
- Sample size will provide 80% power and accounts for 20% attrition, which is estimated from the investigators' preliminary studies.
- Planned outcome measures include both clinical assessments of blood pressure and process measures related to provider acceptance and patient activation. Methods are included to identify barriers and facilitators in the implementation of mGlide in the clinics. Assessments will be available in multiple languages.
- Data management procedures are excellent.
- Statistical analysis plan is strong and appropriate. Sex will be examined as a covariate and potential moderator of the effect of mGlide, so sex as a biological variable is addressed adequately.
- Cost tracking and cost-effectiveness analysis are good.
- Timeline for the research activities is appropriate.

Weaknesses

- There are very limited intervention activities designed to promote use of the mGlide information by providers and other clinic staff. While the project will provide some information on provider acceptability, it will provide far less information on implementation in systems where research personnel (i.e., study nurses and pharmacy experts) are not available through the study. (minor)

- The investigators should estimate the intra-class correlation within provider/clinics even though they make the case to not conduct group randomization by provider or clinic. (minor)
- Power calculations are not provided for analysis of provider-level data. (minor)

5. Environment:

Strengths

- The environment at the University of Minnesota has the resources and facilities needed to perform the proposed research.
- The partnerships with the three health clinics are well-established and will provide the clinical environment and patient population required for the study.

Weaknesses

- None noted.

Protections for Human Subjects:

Acceptable Risks and/or Adequate Protections

Data and Safety Monitoring Plan (Applicable for Clinical Trials Only):

Acceptable

Inclusion of Women, Minorities and Children:

- Sex/Gender: Distribution justified scientifically
- Race/Ethnicity: Distribution justified scientifically
- For NIH-Defined Phase III trials, Plans for valid design and analysis: Scientifically acceptable
- Inclusion/Exclusion of Children under 18: Excluding ages <18; justified scientifically

Vertebrate Animals:

Not Applicable (No Vertebrate Animals)

Biohazards:

Not Applicable (No Biohazards)

Resubmission:

- The applicants have been very responsive to the prior critiques and improved the application.

Applications from Foreign Organizations:

Not Applicable (No Foreign Organizations)

Select Agents:

Not Applicable (No Select Agents)

Resource Sharing Plans:

Acceptable

Authentication of Key Biological and/or Chemical Resources:
Not Applicable (No Relevant Resources)

Budget and Period of Support:
Recommend as Requested

THE FOLLOWING SECTIONS WERE PREPARED BY THE SCIENTIFIC REVIEW OFFICER TO SUMMARIZE THE OUTCOME OF DISCUSSIONS OF THE REVIEW COMMITTEE, OR REVIEWERS' WRITTEN CRITIQUES, ON THE FOLLOWING ISSUES:

PROTECTION OF HUMAN SUBJECTS: ACCEPTABLE

INCLUSION OF WOMEN PLAN: ACCEPTABLE

INCLUSION OF MINORITIES PLAN: ACCEPTABLE

INCLUSION OF CHILDREN PLAN: ACCEPTABLE

COMMITTEE BUDGET RECOMMENDATIONS: The budget was recommended as requested.

Footnotes for 1 R01 HL138332-01A1; PI Name: LAKSHMINARAYAN, KAMAKSHI

Ad hoc or special section application percentiled against "Total CSR" base.

NIH has modified its policy regarding the receipt of resubmissions (amended applications). See Guide Notice NOT-OD-14-074 at http://grants.nih.gov/grants/guide/notice-files/NOT-OD-14-074.html. The impact/priority score is calculated after discussion of an application by averaging the overall scores (1-9) given by all voting reviewers on the committee and multiplying by 10. The criterion scores are submitted prior to the meeting by the individual reviewers assigned to an application, and are not discussed specifically at the review meeting or calculated into the overall impact score. Some applications also receive a percentile ranking. For details on the review process, see http://grants.nih.gov/grants/peer_review_process.htm#scoring.

MEETING ROSTER

The roster for this review meeting is displayed as an aggregated roster that includes reviewers from multiple CSR Special Emphasis Panels of the Health Care Delivery and Methodologies Research for the 2018/01 council round.
This roster for CSR is available at:
http://public.era.nih.gov/pubroster/Reports?DOCTYPE=SEP&DESFORMAT=PDF&AGENDA_SEQ_NUM_P=333326
